# Prevalence and risk factors for hypokalemia in patients scheduled for laparoscopic colorectal resection and its association with post-operative recovery

**DOI:** 10.1186/s12876-018-0876-x

**Published:** 2018-10-19

**Authors:** Qianqian Zhu, Xianlong Li, Fang Tan, Yingqing Deng, Chulian Gong, Jingping Hu, Pinjie Huang, Shaoli Zhou

**Affiliations:** 1 Department of Anesthesiology, The Third Affiliated Hospital of Sun Yat-Sen University, 600th Tianhe Road, Tianhe District, 510360 Guangzhou City, People’s Republic of China; 2 Department of Anesthesiology, The Seventh Affiliated Hospital of Sun Yat-Sen University, Shenzhen City, People’s Republic of China

**Keywords:** Hypokalemia, Gastrointestinal preparation, Risk factors

## Abstract

**Background:**

Perioperative serum potassium levels are closely associated with postoperative clinical outcomes after gastrointestinal surgery. The aim of our retrospective study was to identify the prevalence and risk factors for preoperative hypokalemia (before pneumoperitoneum) and to evaluate the influence of preoperative hypokalemia on the recovery of postoperative gastrointestinal function.

**Methods:**

In this retrospective study, patients scheduled for laparoscopic colorectal resection from November 11 2014 to October 20 2016, were considered for inclusion. A blood potassium level between 3.5 and 5.5 mmol/L was defined as normal, with levels between 3.0 to 3.5 mmol/L, 2.5 to 3.0 mmol/L and < 2.5 mmol/L considered as slight, moderate, and severe level of hypokalemia. The factors including age, gender, ASA grade, BMI, hypertension, diabetes, anti-hypertension drugs, lactose oral soluble, oral cathartics, oral cathartics, cathartic enemas, and blood potassium level before gastrointestinal preparation which might be associated with blood potassium level before pneumoperitoneum were analysed. The time to postoperative first flatus (FFL) and first feces (FFE) was compared between patients with and without hypokalemia.

**Results:**

The final analysis was based on the data of 108 patients. Hypokalemia was identified in 70.37% patients, with the following distribution of blood potassium levels before pneumoperitoneum: slight, 49 (45.37%) patients; moderate, 23 (21.30%); and severe, 4 (3.70%) patients. Hypokalemia was significantly associated with hypertension and the use of ≥2 types of oral cathartics for preoperative gastrointestinal preparation. With treatment, potassium levels recovered to normal levels in all patients within 48 h postoperatively. Hypokalemia was associated with a longer postoperative time to first feces, compared to patients with a normal potassium level before pneumoperitoneum.

**Conclusions:**

Our findings underlie the importance of early monitoring and management of serum potassium levels in these patients.

## Background

Perioperative (including before,during and after surgery) serum potassium levels are closely associated with postoperative clinical outcomes after gastrointestinal surgery [1], including the recovery of gastrointestinal function, the risk for acute kidney injury and adverse cardiovascular events, as well as in-hospital mortality [2–4]. Hypokalemia occurs frequently, being identified in over one fifth of hospitalized patients [5].

Several factors are believed to be involved in the development of hypokalemia [6], including inadequate intake, excessive potassium loss and impairment in the distribution mechanisms of potassium. Although diuretic therapy is considered to be the main risk factor for hypokalemia, diarrhea, vomiting and gastrointestinal preparation prior to surgery may also be contributing factors [7]. For gastrointestinal preparation prior to abdominal surgery, dietary restrictions, oral cathartics, cathartic enemas, and colon-cleansing agents are used to achieve ideal preparation for both surgery and anesthesia. As a result, perioperative hypokalemia is a common finding among these patients, which could affect postoperative gastrointestinal function [8, 9]. However, the prevalence and risk factors for perioperative hypokalemia in these patients remains unclear, including the influence of preoperative (before pneumoperitoneum) hypokalemia on the postoperative recovery of gastrointestinal function. Therefore, the aim of our retrospective study was to identify the prevalence and risk factors for preoperative hypokalemia and to evaluate the influence of preoperative hypokalemia on the recovery of postoperative gastrointestinal function.

## Methods

### Patients and data collection

From November 11 2014 to October 20 2016, a total of 122 patients scheduled for laparoscopic colorectal resection at the Third Affiliated Hospital of Sun Yat-sen University were considered for inclusion. The clinical data of prospective patients was reviewed from medical records to include patients ≥18-years-old, with an American Society of   Anesthesiologists (ASA) Physical grade I/II/III physical status. Patients with a history of familial hypokalemia, pre-existing kidney disease and whose blood potassium levels were not obtained before pneumoperitoneum were excluded.

### Ethical standard

This study was approved by the Ethics Committee of the Third Affiliated Hospital of Sun Yat-sen University and was carried out in compliance with the Helsinki Declaration. The requirement for informed consent was waived because of the retrospective nature of the study and our use of anonymized data.

### Criteria of hypokalemia

In agreement with previous studies, a blood potassium level between 3.5 and 5.5 mmol/L was defined as normal, with levels between 3.0 to 3.5 mmol/L, 2.5 to 3.0 mmol/L and < 2.5 mmol/L considered as slight, moderate, and severe level of hypokalemia [8].

### Statistical analysis

Quantitative variables with a normal distribution are reported as the mean ± standard deviation (SD). The one-sample Kolmogorov-Smirnov Test was used to test the normality of the distribution of quantitative data, the Student’s *t*-test was used to compare: normally distributed variables between the two groups, and the Mann–Whitney U test was used for non-normally distributed data. Categorical data were compared using the chi-squared or Fisher’s exact tests. A multivariable logistic regression analysis (forward regression:LR method) was used to determine risk factors for hypokalemia. Differences were considered significant when the two-tailed *p* values were < 0.05. SPSS 19.0 software (SPSS Inc., Chicago, IL) was used to perform statistical analyses.

## Results

**Fig. 1:**
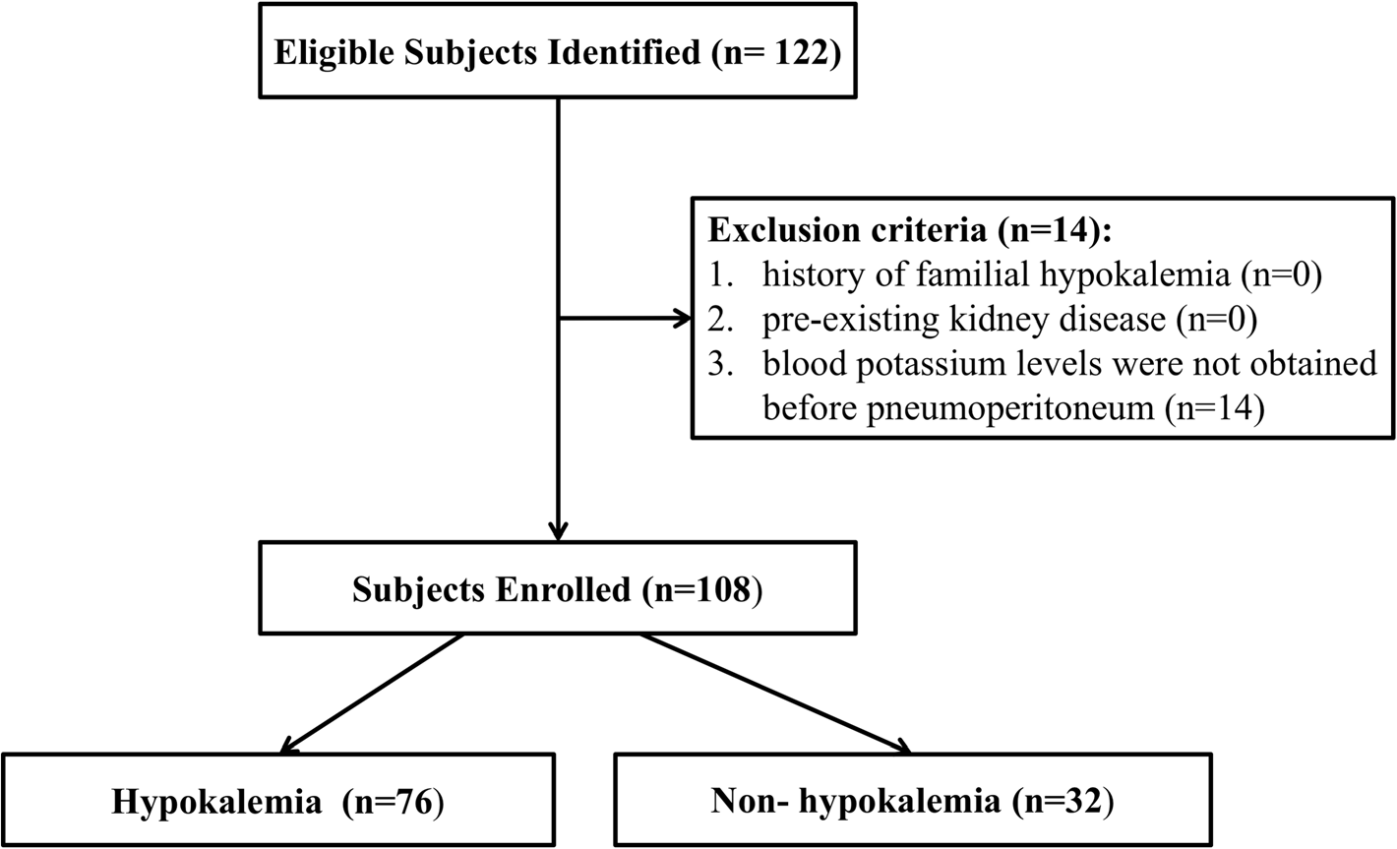
The flow chart of inclusion and exclusion process

The final analysis was based on the data of 108 patients, with at least one blood potassium level obtained during the peri-anesthesia period (Fig. 1). There were 76 patients with hypokalemia. Hypokalemia was identified in 70.37% patients, with the following distribution of blood potassium levels before pneumoperitoneum: slight, 49(45.37%) patients; moderate, 23(21.30%); and severe, 4 (3.70%) patients. The distribution of hypokalemia severity at the different time points of measurement is summarized in Table 1. To identify the preoperative factors associated with hypokalemia before pneumoperitoneum, the 108 patients were classified into the normal potassium level group (non-hypokalemia group) or the hypokalemia group. The characteristics of the 108 patients are summarized in Table 2.

**Table 1:** The distributions of hypokalemia in different time points

	Before gastrointestinal preparation (108)	Before pneumoperitoneum (108)	24 h after surgery (108)
Slight hypokalemia	18 (16.67%)	49 (45.37%)	6 (5.56%)
Moderate hypokalemia	0	23 (21.30%)	0
Severe hypokalemia	0	4 (3.7%)	0

**Table 2:** Characteristics of 108 patients

Characteristic	Hypokalemia (*n* = 76)	Non- hypokalemia (*n* = 32)	*P*-value
Age	60.34 ± 9.89	57.75 ± 13.13	0.107
Gender (female)	41(53.9%)	11(34.4%)	0.063
BMI (kg/m^2^)	22.93 ± 3.26	22.48 ± 2.92	0.616
ASA grade			0.391
I	5(6.6%)	1(3.1%)	
II	58(76.3%)	28(87.5%)	
III	13(17.1%)	3(9.4%)	
Hypertension	34(44.7%)	6(18.8%)	0.011
Anti-hypertension drugs			
Calcium channel blockers	16	2	
β-receptor blocker	3	1	
ACEI and ARB	2	0	
Thiazide	2	0	
Diabetes	9(11.8%)	3(9.4%)	0.97
Lactose oral soluble	30(39.5%)	9(28.1%)	0.262
Oral cathartics (≥2 types)	48(63.2%)	11(34.4%)	0.006
Cathartic enemas (≥2 types)	47(61.8%)	19(59.4%)	0.810
Hypokalemia before gastrointestinal preparation	15(19.7%)	3(9.4%)	0.187
Time to first flatus (h)	58.38 ± 26.05	55.53 ± 27.34	0.618
Time to first feces (h)	97.71 ± 50.83	79.81 ± 37.04	0.045

Notes: Data were presented by mean ± SD and percentages. The one-sample Kolmogorov-Smirnov Test was used to test the normality of the distribution of quantitative data. Normally distributed variables were compared using Student’s *t*-test, non-normally distributed variables using Mann–Whitney U test, and categorical data using the chi-squared or Fisher’s exact tests; *P*-value < 0.05 was considered significant

Abbreviations: *BMI* body mass index, *ASA* American Society of Anesthesiologists, *ACEI* angiotensin converting enzyme inhibitors, *ARB* angiotensin receptor blocker

There were no significant differences between patients with or without hypokalemia with regard to age, sex, body mass index (BMI), and ASA grade. However, the incidence rate of hypertension was greater in the hypokalemia (44.7%) than the non-hypokalemia (18.8%) group (*p* = 0.011). In terms of gastrointestinal preparation, the proportion of patients in whom ≥2 types of oral cathartics were used was greater in the hypokalemia (63.2%) than that in the non-hypokalemia (34.4%) group (*p* = 0.006). With regard to the use of cathartic enemas, although more patients used lactose oral soluble or ≥ 2 types of enemas than in the non-hypokalemia group, this between-group difference was not significant. The variables which were statistical different between hypokalemia and non-hypokalemia group were included in multivariable model. The multivariable logistic regression analysis (forward regression: LR method) was used to determine risk factors for hypokalemia. On multivariable logistic regression analysis, hypertension (odds ratio (OR), 4.067; 95% confidence interval (CI), 1.434–11.532) and use of ≥2 types of oral cathartics (OR, 3.743; 95%CI, 1.508–9.296) were significantly related with hypokalemia prior to pneumoperitoneum (Table 3). Following intraoperative treatment, only 6 patients presented with slight hypokalemia at 24 h post-surgery (Table 1).

**Table 3:** Multivariable logistic regression results for Hypokalemia patients

Factors	OR	95% CI		*P*-value
		Lower	Upper	
Hypertension	4.067	1.434	11.532	0.008
Oral Cathartics (≥2 types)	3.743	1.508	9.296	0.004

Notes: Multivariable logistic regression analysis (forward LR method) was used to determine risk factors for hypokalemia; *P* < 0.05 was considered to be statistically significant

Abbreviations: *OR* odds ratio, *CI* confidence interval

With regard to the association between blood potassium level prior to pneumoperitoneum and postoperative gastrointestinal function, the time to postoperative first flatus was not significantly different between patients with a normal blood potassium level (55.53 ± 27.34 h) and those with hypokalemia (58.38 ± 26.05 h, *p* = 0.618). However, the time to first feces was significantly longer among patients with hypokalemia (97.71 ± 50.83 h) than in those with a normal blood potassium level (79.81 ± 37.04 h, *p* = 0.045).

## Discussion

In our study group, the incidence rate of hypokalemia was 70.37%, with 3.70% patients presenting with severe hypokalemia in the peri-anesthesia period. Hypokalemia was significantly associated with hypertension and the use of ≥2 types of oral cathartics for preoperative gastrointestinal preparation. With treatment (potassium chloride was intravenous infusion until potassium level reach 3.5 mmol/L during surgery, oral potassium 1 g/day in inpatient ward), potassium levels recovered to normal levels in all patients within 48 h postoperatively. Hypokalemia was associated with a longer postoperative time to first feces, compared to patients with a normal potassium level before pneumoperitoneum.

The prevalence of rate of hypokalemia in our study group of 70.37% was higher than the rate previously reported for a general group of hospitalized patients [5]. Hypokalemia is common in hospitalized patients. Various factors can contribute to hypokalemia, including insufficient intake, excessive loss of potassium and inadequate potassium distribution. Specifically for laparoscopic resection, the strict gastrointestinal preparation required can lead to hypokalemia. Our findings are in agreement with a previous study which demonstrated that the development of hypokalemia was a common complication of polyethylene glycol-based preoperative bowel preparation [10]. Though the polyethylene glycol is the most frequently used solutions for bowel preparation, other oral cathartics were also used. In the present study, patients with constipation were more prone to take other cathartics like lactulose or mannitol before bowel preparation with polyethylene glycol, even early when admitted to hospital. Mannitol administration could also induce hypokalemia [11]. We also identified that patients in whom ≥2 types of oral cathartics were used were more likely to develop hypokalemia. Therefore, potassium monitor should be recommended for these patients early even before gastrointestinal preparation.

In addition to potassium loss through the gastrointestinal tract, the use of diuretics can further contribute to hypokalemia [12]. Thiazide, which is often prescribed to patients with cardiovascular disease, such as hypertension, can induce hypokalemia through this mechanism [13]. In our study, the prevalence of hypertension was greater among patients with hypokalemia than without. However, the use of anti-hypertension drugs, including thiazide, was comparable between the two groups, a finding which might be attributed to the limited number of patients. Of note, however, hypertension was identified as an independent risk factor for hypokalemia on multivariable analysis in our study. Hypokalemia in hypertension might result from an increase in urinary loss of potassium with the use of anti-hypertensive drugs, such a thiazide mentioned above, which are used to block the increased reabsorption of sodium associated with hypertension [14]. Of note, although hyperkalemia has been described as an adverse outcome of the use of angiotensin-converting-enzyme inhibitors (ACEIs) and angiotensin receptor blockers (ARBs), two types of anti-hypertensive drugs, hypokalemia has also been described, particularly in those patients on dual therapy [15].

Although hypokalemia is often asymptomatic, it remains a risk factor of gastrointestinal disorders and serious perioperative and postoperative arrhythmia [16–19]. Hypokalemia might also contribute to delayed recovery from anesthesia [20], as well as increasing the length of hospital stay and the risk for all-cause and cardiovascular-related mortality [5, 21]. Among our study group, the time to first feces was significantly longer among patient with than without hypokalemia. Therefore, maintaining normal potassium after laparotomy would be important to lower the risk for gastrointestinal mortality and to improve postoperative recovery. In fact, in the “enhanced recovery after surgery” (ERAS) protocol, prophylactic potassium administration is recommended [22]. Another study even demonstrated that the prevention of hypokalemia prior to admission was effective in enhancing the recovery of patients undergoing open abdominal surgeries [8].In our study group, the prevalence of hypokalemia prior to gastrointestinal preparation was not high (16.67%), with the level of hypokalemia in these patients being slight. By contrast, the prevalence of hypokalemia increased to 70.37% after gastrointestinal preparation, with 3.70% of patients presenting severe hypokalemia. Previous studies demonstrated that early serum potassium monitoring allowed for early correction of hypokalemia and effective recovery from surgery [4, 8]. Based on this evidence, early potassium administration has been strongly recommended for patients scheduled for elective laparoscopic colorectal resection under strict monitoring, especially during the period of bowel preparation. Whether hypertensive patients were more like to develop hypokalemia during gastrointestinal preparation remains to be confirmed and explored in future studies.

There are several limitations of the present study which need to be declared. First, because of the limited number of patients and selection from a single center, results, including the prevalence of peri-anesthesia hypokalemia, will need to be confirmed in larger, multi-center, cohort studies. Second, due to the retrospective nature of our study, reasons for different bowel preparation used were not clear, and might have biased our results. Third, detailed information, such as the oral intake of potassium and ambulation status of patients, could not be extracted from medical records and, again, might have biased our results. Fourth, the parameters including the time to postoperative first flatus and the time to first feces used to determine the gastrointestinal motility are clinical assessment but not the golden standard. Scintigraphic recording should be used in future study.

## Conclusions

Our study demonstrated the high prevalence of hypokalemia and severe hypokalemia during the peri-anesthesia period in patients who underwent laparoscopic colorectal resection. Hypokalemia before pneumoperitoneum was associated with a longer time to first feces postoperatively. Our findings underlie the importance of early monitoring and management of serum potassium levels in these patients.

## Abbreviations

ACEIs: angiotensin-converting-enzyme inhibitors
ARBs: angiotensin receptor blockers
ASA: American Society of Anesthesiologists
BMI: body mass index
CI: confidence interval
ERAS: enhanced recovery after surgery
FFE: first feces
FFL: first flatus
OR: odds ratio
SD: mean ± standard deviation
